# Experience of Wellness Recovery Action Planning in Self-Help and Mutual Support Groups for People with Lived Experience of Mental Health Difficulties

**DOI:** 10.1155/2013/180587

**Published:** 2013-01-09

**Authors:** Rebekah Pratt, Andy MacGregor, Susan Reid, Lisa Given

**Affiliations:** ^1^Department of Family Medicine and Community Health, University of Minnesota, 717 Delaware Street, Minneapolis, MN 55414, USA; ^2^Scottish Centre for Social Research, Edinburgh EH3 9AW, UK

## Abstract

The main aim of this research was to assess the relevance and impact of wellness recovery action planning (WRAP) as a tool for self-management and wellness planning by individuals with mental health problems from pre-existing and newly formed groups, where the possibilities for continued mutual support in the development of WRAPs could be explored. Interviews and focus groups were conducted and pre-post recovery outcome measures completed (Recovery Assessment Scale and Warwick Edinburgh Mental Well Being Scale). 21 WRAP group participants took part in the research. The WRAP approach, used in groups and delivered by trained facilitators who could also share their lived experience, was very relevant and appeared to have a positive impact on many of the participants. The impact on participants varied from learning more about recovery and developing improved self-awareness to integrating a WRAP approach into daily life. The apparent positive impact of WRAP delivered in the context of mutual support groups indicates that it should be given serious consideration as a unique and worthwhile option for improving mental health. WRAP groups could make a significant contribution to the range of self-management options that are available for improving mental health and well-being.

## 1. Introduction

### 1.1. Self-Help and Recovery

There has been a growing commitment to a recovery approach in mental health in the recent years in many countries [1]. This has been particularly evident in Scotland, and the Scottish Recovery Network has supported a wide range of recovery-promoting activities [2–6]. Alongside this interest in recovery, there has been an increasing emphasis on self-help, self-management, and mutual support as important options in the range of therapeutic encounters that people seek to address mental health concerns [7–13]. Self-help and self-management approaches to mental health vary in their approaches but offer a potentially consistent way to explore key concepts of recovery, particularly those of personal responsibility, education, hope, self-advocacy and support, through patient self-directed interventions.

There are a range of self-management approaches available for people to enhance well-being, from guided self-help, such as computerized CBT [14, 15], mindfulness [16], and bibliotherapy [17, 18]. However many self-help or self-management interventions are professionally developed resources, which may focus on education and personal responsibility but have the potential to be promote adherence with professional-led views on mental health, rather than encouraging patient-led perspectives. Some approaches, such as the recovery workbook approaches [19–21], draw on partnerships between lay people and professionals to offer self-management strategies. Self-management approaches that have been developed by nonprofessionals, or people with lived experience, also exist, such as peer support [5]. One more formalized version of self-management that emerges from the experience of lay people is wellness recovery action planning, or wRAP. WRAP was developed in the USA by a user of mental health services, Mary Ellen Copeland, and as such is a nonprofessionally developed recovery approach based on self-management approach to improve mental health and well-being [22].

### 1.2. Wellness Recovery Action Planning (WRAP)

WRAP explores key values of recovery, such as hope, personal responsibility, education, self-advocacy, and support, and provides a structured process for developing individualized WRAP plans [22]. These individualized WRAPs serve to document triggers for difficult feelings or behaviors, encourage the identification of tools that contribute to well-being, propose ways to self-monitor, help develop action plans for managing wellness, and can be plans that are shared with others should that be necessary in times of illness or crisis [23]. The focus of self-management resonates closely with the recovery approach, particularly in relation to empowering people to manage their own health and conditions. 

WRAP is reported in the research to be perceived positively by those who have used it. The findings from the research on WRAP that have been conducted tend to elicit very positive feedback from those that attend a WRAP group [24–26]. WRAP has also been reported as being particularly useful for the identification of triggers for negative mental health [26, 27] and for developing daily strategies for wellness [25–27]. Studies have also demonstrated an increase in expressing hope for recovery, taking responsibility, having a support system in place, managing medications, and developing a crisis plan [27]. Of those studies that were conducted in group settings, there appeared to be much positive feedback about the role of mutual support [24, 26]. 

WRAP has also been shown to have a positive impact on mental health outcomes. Two studies with much larger sample sizes found that following the use of WRAP, there were significant decreases in global symptom severity, a statistically significant decrease in symptoms, and a significant increase in the scores for recovery [23, 28]. A sample of 58 consumers undertaking WRAP groups showed a reduction in psychiatric symptoms and an increase in hopefulness in comparison with those who did not receive WRAP [29]. A randomized study in Ohio, which randomized 519 participants, showed reduction in anxiety and depression and an improvement in Recovery Assessment Scale scores [30]. Two further studies also indicated that participants described changing behaviors to support wellness [24, 27], and reported an improvement in quality of life [26]. 

The benefits of WRAP extend to how individuals might also interact with others around them, such as carers or professionals. Research that focused on self-reports showed that participants reported an increase in more positive thinking [26], greater confidence in talking to doctors about their needs [25, 26], and improved ability to seek and get support from family, professionals, and others in the WRAP group [26, 31]. There was an increased knowledge of recovery, and this appeared to have a very empowering and inspirational impact on participants [32]. These benefits of better engaging with others have important implications for self-management, showing the potential benefits of successful self-management leading to fostering better patient-led care and planning.

WRAP has been used in a number of settings and cultures, such as with Chinese mental health consumers in New Zealand [26] Somali women in England [33], and Black and minority ethnic women in Glasgow [24]. These studies have reported positive experiences for participants of WRAP although there was some indication that the WRAP approach could be better adapted to be more culturally appropriate to different groups. The participants in one study included people with lived experience of mental health problems, practitioners, and carers, illustrating the potential usefulness of WRAP to be conducted in mixed groups [32].

### 1.3. WRAP in Scotland

The Scottish Recovery Network supported four organizations throughout Scotland to train people with lived experience of mental health problems as WRAP facilitators, who would go on to run WRAP groups in their own organisations for people who were having mental health difficulties. Each organization identified two facilitators who were given advanced level training as WRAP facilitators and who then went on to deliver WRAP groups. This research followed the delivery of WRAP for the first two groups (often also described as courses or as training) undertaken by the newly trained facilitators. WRAP can be delivered in a variety of formats, but for this intervention, all were delivered in groups. During the first and second deliveries of WRAP, two of these organisations convened the WRAP training over two days whereas the other two organisations spread the WRAP training over 4 days. Both groups who conducted the training over 4 days ran the sessions a week apart over 4 weeks. One group that ran two sessions did so a week apart for both the first and second deliveries of training, and the other ran the first training delivery one week apart and the second delivery on two consecutive days over a weekend.

The main aim of this research was to assess the relevance and impact of wellness recovery action planning (WRAP) as a tool for self-management and wellness planning by individuals with mental health problems in mutual support group settings.

The specific objectives of the research we report on here are as follows.

1. An assessment of the extent to which participants who received training benefited in terms of recovery and wellness and the extent to which they used their own WRAP to help them do so.
2. An examination of the role of self-help and mutual support groups in supporting recovery and wellness planning.

In this paper we report on the aims as they relate to the groups' participants. Data describing the experience of the facilitators has been reported elsewhere [34]. 

## 2. Methods

The experience of those taking part in the WRAP training groups was explored in different ways, through focus groups, individual face-to-face interviews, by collecting pre-post test outcome measures, and by the use of session-by-session evaluation forms. Qualitative data was only collected on a sample of participants taking part in the first round of WRAP training, and quantitative data was collected in both rounds of delivering WRAP training. 

The participants who took part in the first WRAP training sessions were invited to attend a focus group at the end of their training. Four focus groups with group participants were conducted in each of the 4 sites between December 2009 and February 2010. In total, 21 people participated in the focus groups. Group participants were also asked to complete brief session-by-session evaluation forms although these were mostly used by the facilitators themselves to gauge the usefulness of their individual training sessions and/or topics covered in the groups, and those are not reported on here.

Follow-up individual face-to-face interviews were conducted with 11 people who took part in the baseline participant focus groups. Participants across all four focus groups, from the four different organizations, were interviewed. These interviews were conducted 3-4 months after the baseline focus group had taken place, in order to explore their experience of developing their own WRAP and whether participants were still using the WRAP approach. 

The use of scales that measure outcomes has increasingly been used to establish the effectiveness of self-management approaches in mental health. This project used two different outcome measures: one that focuses on recovery, the Recovery Assessment Scale, and one that focuses on well-being, the Warwick-Edinburgh Mental Well-being Scale (WEMWBS). Participants completed the short version of RAS and WEMWBS before and after their WRAP training, and these have been used to show any improvements in self-rated mental health following attendance of the WRAP training sessions. 

### 2.1. Analysis

All focus group and interview data were recorded, transcribed, and analyzed in NVivo8 (QSR). A thematic analysis was conducted, following the principles of the social constructivist version of grounded theory [35]. Themes and subthemes were identified and organized as they emerged from the data. Emerging themes were discussed and reviewed regularly by the research team to ensure reliability and validity of the qualitative analysis. The quantitative outcome data were analyzed using Excel; due to the relatively small sample size basic frequencies and means were calculated only. Pre- and postscores of RAS and WEMWEBS were compiled and reported. 

### 2.2. Ethics

The ethical subcommittee of the Scottish Centre for Social Research's parent organization, the National Centre for Social Research, reviewed the study and gave it ethical clearance in July 2009. No National Health Service sites were included in this research and as such an ethical opinion was not required from the NHS. 

## 3. Findings

First we describe the qualitative findings from the WRAP group participants, and then we describe the recovery and well-being measures collected.

### 3.1. Group Participants Experience

Many of the participants described attending the WRAP group as a process to learn about themselves and reflect on the various aspects of their mental health. The benefit of going through that process of reflection was viewed as having the potential to increase self-awareness and acceptance. One of the key benefits for many participants was learning about a recovery approach. For many participants this was a new and different way of looking at their lives, which was transformative for some. Here we describe the reported benefits of WRAP, the impact of the group setting, the levels of integration of WRAP into daily life, and the challenges encountered.

#### 3.1.1. Perceived Benefits of WRAP

The consensus was that there were lasting benefits from undertaking WRAP. These included being able to challenge your own behaviors, identifying alternative responses, and evaluating what constitutes a priority. Participants reported a reduction in anxiety, a reduction in panic attacks, and an increased identification of their own triggers for negative mental health. The perceived benefits of WRAP led to feelings of increased confidence and self-esteem for many. Participants gained insight into triggers for poor mental health and the benefits of identifying wellness-promoting activities. The sense of achievement was evident from being able to find different, useful approaches to dealing with stressful situations. One participant said that she had put her Christmas tree up for the first time in three years and attributed this action to having undertaken the WRAP course. Seeing strategies written out offered confidence and guidance, as participants described they could check back on their own ideas and get clear feedback or reinforcement from their WRAP. These strategies were ones that were tailor made, by the participants, for the participants. This led to a sense of ownership of the WRAP plan.

*Um…it's for me, I think its mine, its nobody else's and I wrote it, I think…that's how I see me. And I think that's probably how a lot of other people see me so it's not something that somebody else has wrote and I need to follow it. It's just…mine I think. Yeah. (Follow-up interview)*

#### 3.1.2. Group Setting

Some respondents reflected that they felt undertaking WRAP in a group, compared with undertaking WRAP one to one, would be more supportive, less intense, and had the potential to offer mutuality and the ability to learn together. Participants described a range of benefits to this, including increased confidence, challenging stigma, increased self-esteem, and a feeling that they were not alone. Participants valued having the opportunity to both offer and receive support.

*Well I feel…I mean I don't talk to strangers about ma condition. It's like I live a secret life, like nobody knows I've got this condition, but when I come here I can talk to people that.. that understand what I've went through and stuff, you know?, and they might come up wi' suggestions aboot maybe medicine they've been o.. You know? You just pick up different ideas. (Focus group)*

Many of the participants had experienced a range of interventions, and some reflected that the collective setting was much more interactive for participants, and that was seen to increase the likelihood of engaging with the approach.

*Whereas on a one-to-one, for a start you'd be embarrassed to ask any questions. You'd just go, “Oh, right you are. Thanks very much”, you know?:… and you would… you wouldn't want to sort o' say, “Well, look. I don't really understand that”. But it was so easy, so everything that anyone has said here, the (facilitators), the charts where all our thoughts went up. (Focus group)*

#### 3.1.3. Integration of WRAP into Daily Life

The vast majority of the participants, both in the focus groups and the follow-up interviews, had drawn on WRAP in their daily lives. In the follow-up interviews, the majority had used their WRAPs and intended to continue to do so. The ways in which participants integrated WRAP into their lives varied at times, and the many different ways in which people utilised their WRAP and the related activities and resultant benefits can be broadly characterised as having drawn on the concept and ideas, engaging in the WRAP process itself, integrating the WRAP into daily life, and fully integrating WRAP into activities and outlook.  Table 1 describes this in further detail.

Not all participants went on to develop their own WRAP. Some comments suggested that undertaking a WRAP was challenging, and actively using the components contained within it required a certain level of commitment and motivation. Some felt that they were not in the right place in their life or feeling well enough to explore the WRAP process further. However, many reflected that the process of systematically working through a process of self-awareness was very helpful, even if they did not develop a WRAP as such. This process alone appeared to offer useful insights into one's own experience of living with a mental illness. 

Almost all of the participants reported that they were drawing on WRAP in their daily lives. For some it provided an element of security, a comforting feeling that there was a way to look at mental health, and tools to draw on that would support recovery. Ultimately, a few participants felt that their WRAP had so significantly influenced their well-being that it had started to become an intuitive way of thinking.

*And I feel that um…in the WRAP training it's almost along those lines of quite fundamental in…um…it's almost a seismic shift in thinking. Um…where there was no thinking, only confusion and mystery before there was great mystery why is this happening to me? (Follow*-*up interview)*

The structured approach of WRAP offered a tool for communicating with others that was manageable and constructive. A few participants had shared their WRAP with care professionals, who had contributed positively to the sense of being involved in the planning of care, including their preferences for care should there be a crisis. Others described having gained the confidence to share their experiences of mental illness with friends or family for the first time, some even describing that they no longer had to live a “secret life” of managing mental illness. 

#### 3.1.4. Challenges

One challenge was that crisis planning aspect could be quite difficult for participants to complete, both because respondents thought that they had either not experienced a genuine “crisis” or because of the sensitivities of thinking back to a time of crisis. Where participants had experienced a crisis that resulted in admission to a health care facility, this part of WRAP was seen as being vital at communicating the individual's wishes to friends, family, and health care staff.

* I wonder if an acceptance of the fact that sometimes your lie will be in crisis, and that knowing that there's another side storm. You come out the storm. (Focus group)*

### 3.2. Recovery Measures for Group Participants

Table 2 shows the scores from the groups of participants for each of the four participating organisations. This shows that RAS scores increased in all groups, and WEMWBS scores in all but one group, after the respondents had completed their WRAP training. This suggests that the participants had more positive views in relation to their own sense of recovery and well-being having been trained in WRAP. However, these results must be treated with caution as the numbers who completed the forms were relatively small and the pre- and post-WRAP training questionnaires were not completed by the same number of people. Any differences between pre- and post-WRAP training scores might, therefore, be due to the low sample size and also that people with higher scores may be more likely to complete the post-WRAP training questionnaires. However, these results do support the very positive views expressed by group participants in the main qualitative phase of the study. Note that the Recovery Assessment Scale ranges between 20 and 100; 20 = very low mood/pessimistic about future and 100 = very optimistic/positive, and WEMWBS ranges between 14 and 70; 14 = very negative views and 70 = very positive views.

## 4. Discussion

This research found that the WRAP approach used in groups and delivered by trained facilitators who could also share their lived experience was very relevant and appeared to have a positive impact on many of the participants. The group-based format and facilitators offering mutual support through sharing their lived experience with group members appeared to be important aspects of the impact of this approach. 

The level of impact varied among the participants, and ranged from increased awareness gained from the concept and ideas of recovery, increased self-awareness, integrating WRAP tools and self-management into daily life, and fully integrating it into their thinking about well-being. All of these levels of impact offered substantial benefits for participants even if participants did not go on to complete their own written version of their WRAP. This is an important point to note in ongoing work researching WRAP completed WRAP plans may not be an indicator of how much the intervention might impact participants.

Most participants had not come across the concept of recovery before this experience and found that this offered a useful, and for some, powerful new perspective on their experience. Participants described feeling they could take ownership over their well-being and were able to challenge stigma to the point where they could talk about their experiences, sometimes for the first time. As put by one participant, WRAP offers a reminder of what you are like when you are well, and that offered hope and uncovered strategies for overcoming challenges when encountering an episode of illness. These findings are consistent with the growing literature on the positive benefits of undertaking WRAP [23, 24, 26, 27]. Additionally, in this research the impact also appeared to have been sustained over time (as illustrated by the 3-4 month follow-up interviews), although it would be worthwhile to gauge this sustainability over a longer follow-up period in the future.

The group setting appeared to provide optimal conditions for the delivery of WRAP. The provision of mutual support appeared to enhance the recovery-orientated principles of WRAP. Mutuality offered a supportive, caring environment, and it was viewed as being particularly positive that facilitators were also able to share their experiences. The group aspect of the WRAP intervention itself was clearly an important factor to the success of the WRAP groups, and research on other group recovery orientated approaches based in group settings have also demonstrated positive benefits for participants [19–21]. It would appear, though, that WRAP does require some local modifications for different contexts. 

There are limitations that should be considered in relation to this research. It may have been the case that the individuals taking part in the study were a fairly self-selecting group, both in terms of participating in the interviews and in the intervention itself. The WRAP program and evaluation only focused on four organizations, and it is possible that other organizations and groups would not have had such a favorable response to WRAP. As the sample size of the study was relatively small, the limited quantitative results should be viewed with some caution, even if they tended to support the positive perceptions elicited during the qualitative research phase. Similarly, whilst there were attempts to collect control group data in this research, and RAS and WEMWBS scales were distributed to those attending self-help groups not using the WRAP approach, there were insufficient returns to include as a comparison. Future research should consider including control group data. Although there was very little indication of negative impact, the potential for a negative impact should continue to be monitored in future work.

The apparent positive impact of WRAP delivered in the context of mutual support groups indicates that it should be given serious consideration as a unique and worthwhile option for improving mental health. WRAP groups could make a significant contribution to improving mental health and well-being, and further research that established and compares its effectiveness with other modalities may be worthwhile. WRAP is a self-management tool that is underpinned by mutuality and empathy, not by a professionally-applied treatment or therapy, and therefore offers a unique alternative to professionally-driven approaches, yet with much consistency with the patient-led emphasis of self-management. The results of the research indicate that WRAP has the potential to offer a unique and useful approach that could play an important role in the development of interventions for improving mental health.

## Figures and Tables

**Table 1:** Levels of integration of WRAP into daily life.

Level of integration	Activities	Example
The concept and ideas	(i) WRAP training as an introduction to the concept of recovery. (ii) Thinking about recovery in relation to own experience of mental illness and mental well-being. (iii) Increased awareness of self and challenging stigma.	*“I always vow never to go back up there (acute inpatient ward), but I end up being back there, and I think I actually have to try and take the control more into my own hands, and I think obviously WRAP is one way that I can take back that control, and.. so it is definitely something that I will get round to doing, because I'm determined that the only way I can feel better is.. with mental illness.. is definitely you really need to take the control because there is no.. there's no answers.” (Follow up interview) *

The WRAP process itself	(i) Process of self-reflection and benefits of mutual support environment. (ii) Mostly using the WRAP itself in the group meetings only. (iii) Increased insight into own mental well-being, including identification of triggers and wellness strategies.	*“I think that the only person can do it is yourself. I think it's got to be in.. from the inside out. I don't see how it can be done from the outside in. D'you understand what I mean?” (Focus group) *

Integrating WRAP into daily life	(i) Continuing to refer to WRAP and using it in daily activities. (ii) Drawing on the learning of WRAP to self-monitor behaviour and thinking. (iii) Using WRAP to guide changes in behaviour to promote well-being.	*“I found it really useful for…like…like if I am…very stressed or whatever, I find it very good. Because when I refer to it, it sort of cheers me up because I think to myself oh well I don't want to end up in hospital again. I want to keep myself well and I refer to it as like I have got to focus on everyday and get up in the morning, and listen to music, do things that make me feel good. So I look at it in a positive light.” (Follow up interview) *

Fully integrated WRAP	(i) Using WRAP regularly. (ii) WRAP becoming integrated to a point where it feels it becomes an intuitive way of looking at your life. (iii) Telling others about WRAP as a concept. Sharing own WRAP with others.	*“But for me, it's given me.. it's given me a better understanding of my own mental health and my mental health state. But no' only that, it's given me confidence in myself, you know,.. you know, that I've gained throughout the group and the Support Workers. The.. there is light at the end o' the tunnel. But it's also given that same confidence to my family because they've had the benefit from the WRAP as well. It's not just me that's, you know, that's getting the benefit from it. My family's getting that as well because they can see the difference. It's like, you know, my eldest daughter said, “My dad's back”, and that's how she explains it.” (Focus group) *

**Table 2:** Groups one and two participant responses to scales pre- and post-WRAP training.

Participants	Recovery assessment scale		WEMWBS	
	Pre-WRAP	Post-WRAP	Pre-WRAP	Post-WRAP
Site 1 group 1	64.6 (*n* = 9; 51–74)*	76.4 (*n* = 7; 61–96)	39.6 (*n* = 9; 21–51)	46.1 (*n* = 7; 30–62)
Site 1 group 2	67.5 (*n* = 8; 39–82)	70.0 (*n* = 6; 38–92)	39.0 (*n* = 6; 27–46)	40.7 (*n* = 6; 22–55)
Site 2 group 1	62.3 (*n* = 3; 56–72)	74.0 (*n* = 4; 62–87)	38.0 (*n* = 4; 21–48)	40.8 (*n* = 4; 31–51)
Site 2 group 2	59.8 (*n* = 4; 56–62)	Not available	35.75 (*n* = 4; 31–42)	Not available
Site 3 group 1	83.1 (*n* = 8; 74–99)	87.3 (*n* = 7; 79–100)	48.8 (*n* = 8; 35–63)	54.0 (*n* = 7; 46–61)
Site 3 group 2	67.9 (*n* = 7; 51–92)	75.4 (*n* = 5; 60–94)	42.5 (*n* = 6; 27–53)	48.0 (*n* = 5; 30–67)
Site 4 group 1	83.5 (*n* = 2; 75–92)	92.5 (*n* = 2; 89–96)	60.0 (*n* = 2; 58–62)	51.7 (*n* = 3; 43–57)
Site 4 group 2	73.3 (*n* = 7; 46–89)	82.1 (*n* = 7; 69–99)	44.3 (*n* = 8; 16–53)	49.3 (*n* = 8; 25–62)

Note to tables: *Mean score presented for all participants combined; range of responses included in brackets.

Only one RAS questionnaire was returned following the second group for site 2 WRAP training group. These data are not included in the table as it cannot represent the scores of the whole group.

Only one WEMWEBS questionnaire was returned following the second group of site 2 WRAP training group. These data are not included in the table as it cannot represent the scores of the whole group.
